# Dual-Color Lasing Lines from EMPs in Diluted Magnetic Semiconductor CdS:NiI Structure

**DOI:** 10.34133/2019/6956937

**Published:** 2019-11-05

**Authors:** Muhammad Arshad Kamran, Bingsuo Zou, Kang Zhang, Xiongtao Yang, Fujian Ge, Lijie Shi, Thamer Alharbi

**Affiliations:** ^1^Department of Physics, College of Science, Majmaah University, Al-Majmaah 11952, Saudi Arabia; ^2^Key Lab of Featured Metal Resources Utilization and Advanced Materials Development, Nano and Energy Research Center, School of Physics, Guangxi University, Nanning 530004, China; ^3^Beijing Key Laboratory of Nanophotonics & Ultrafine Optoelectronic Systems, Beijing Institute of Technology, Beijing 100081, China

## Abstract

Have one ever seen a semiconductor that can issue two-color lasing lines? The diluted magnetic semiconductor (DMS) can do this. Here, we have observed dual lasing lines of 530 nm and 789 nm from a DMS structure of CdS:NiI, in which the excitonic magnetic polaron (EMP) and localized excitonic magnetic polaron (LEMP) are excitations out of ferromagnetic (NiS)_*x*_ nanocluster and NiI_2_ nanoclusters within CdS lattice; both of them could lead to the collective EMP state at high excitation and therein produce coherent emission lines simultaneously. This occurrence is due to the superposition of EMP near CdS bandedge and the combination of the charge-transfer band of (NiI)_*n*_ cluster with the LEMP within CdS lattice by overcoming the strong electron correlation of NiI cluster in a DMS structure, evidenced also by ab initio calculation. This finding opens a way to understand the collective behaviour of spin-coupled excitons in DMS and to find novel applications in the spin-related quantum technology.

## 1. Introduction

Lasing from semiconductor structures often comes from the exciton-exciton interactions [1], electron-hole plasma [2], and exciton-polaritons [3] near their bandedge, whose advancement makes modern technology powerful and people's lives colorful. However, no one has ever detected dual lasing lines with large-separation issuing from one semiconductor structure at the same time because there are no different dense excitons of very different energy values except those semiconductor nanostructures in the optical cavity [4, 5]. In contrast to the photon binding in an optical cavity, the local spontaneously ferromagnetic moments or coupled spins in a DMS structure supplied another choice to bind excitons to the collective EMP formation for single-mode lasing [6, 7].

DMSs usually have the transition metal ion dopants and spin-dependent transport and magnetism [8, 9]; however, their spin-related optical properties in bulk have seldom been studied except for their QDs [10, 11] and QWs [12, 13] because the incorporation of transition metal ions into semiconductor lattice could not produce a very uniform phase in macroscopic scales [14]. For transport of the carrier, as the fermion, different energy values will always interact with each other in the near area with the Pauli exclusion principle. For photons with different energies or modes, as a boson, their varied modes may dominate and propagate in the same area at the same time in semiconductor optical cavity due to different excitons therein [5]. This is just like the excitons bound by the specific photon with fixed polarization in the microcavity, in which exciton coupling with photon can lead to exciton-polariton in the coupled exciton lattice structure [15] with potential multiline lasing. In the same way, the excitons can also bind with other bosonic excitations like coupled spins in nanostructures. Can the composites of EMPs behave like the cavity photons binding with more excitons to produce different lasing lines? Until now, such lasing multilines by EMPs still did not come up in a semiconductor structure at the same time with minor temperature dependence.

For a long time, scientists focus on achieving multicolor/tunable laser from a single semiconductor nanostructure for the potential applications in biological inspection [16], full-color laser display [17], optical communication [18], and high-efficiency solar cells. Tuning of lasing over the broad spectral-wavelength is fundamentally a critical issue for several reasons [19–21]. One of the key reasons is the limitation of gain bandwidth (<30 nm) in the structure under the excitation mode. It restricted the tunability of lasing emission. This issue has been dealt with different complex growth methods, *i.e.*, growth of ternary/quaternary alloy and core-shell heterostructures [5–7]. It always remains very difficult to grow such crystal due to the large lattice mismatch with varied compositions. Even though some successes have been achieved to grow such nanostructures with variable lasing lines at different locations, it is still a challenge to grow a structure which can give dual lasing lines with more than 200 nm span at the same location.

II-VI semiconductor (binary, ternary, and quaternary) nanostructures are very attractive for light-emitting diodes (LEDs) and multicolor/tunable bandedge lasing applications. Liu et al. synthesized Zn_*x*_Cd_1‐*x*_S nanoribbons and obtained 40 nm tunable lasing emission from 340 to 390 nm and 30 nm tunable lasing emission from 485 to 515 nm, respectively. Liu et al. [22] reported 62 nm (from 578 to 640 nm) tunable lasing emission in CdSSe alloy [23]. In the same alloy, Liu et al. tuned lasing emission up to 107 nm [24]. All the above-mentioned advances signify the importance of CdS-based nanostructures.

CdS have a direct bandgap of *E*_g_ (wurtzite) = 2.42 eV at *T* = 300 K and have a large exciton combination energy (28 meV). Agarwal et al. pointed out that CdS nanostructures are good optical waveguides and can function as highly optical confined cavities for lasing [25]. Owing to this property, lasing has been observed in CdS nanostructures including nanowires (NWs) [26, 27], nanoribbons (NRs) [28], and nanobelts (NBs) [29]. Their lasing lines only occur at around their bandedge, originating from the exciton effect or electron-hole plasma which never moved to the near-infrared (NIR) zone.

Doping CdS with transition metal ions (TM) has achieved great success in tuning the d-d radiation emission in its 1D nanostructures [30]. However, not a single attempt has been reported in which dopants played a vital role in tuning lasing lines in their DMS structures [7, 30–32]. In this article, we have doped different Ni(II) compounds like NiCl_2_, NiF_2_, NiBr_2_, and NiI_2_ into CdS NBs and explored their optical properties. The crystallinity of as-doped NBs and successful incorporation of Ni ions were confirmed by EDX, XRD, and Raman spectroscopy. As in the case of NiCl_2_, and NiF_2_ doping, NBs grow laterally and almost without F and Cl incorporation; however, NiBr_2_ dopant turns NBs semivertically with some Br incorporation. But with NiI_2_ doping, NBs grow vertically along the substrate with Ni(II) and I^−^ incorporated. The CdS NB prepared with NiCl_2_, NiF_2_, and NiBr_2_ showed similar PL emissions in the range of 756–766 nm due to the d-d transition (^3^T_1g_⟶^3^A_2g_) of Ni ions [32, 33], but this transition was not clearly identified in the PL spectra of NiI_2_-doped NBs. On excitation with fs 405 nm, NiI_2_-doped NB showed lasing lines at 530.9 and 789.3 nm simultaneously, in contrast to that with one line in NiF_2_-, NiCl_2_-, and NiBr_2_-doped NBs. The dual-color lasing lines with a wavelength span of 258.4 nm were detected in this doped CdS NB for the first time. Unveiling the nature of such a phenomenon in CdS:NiI becomes important and urgent for new lasing material design.

## 2. Results

### 2.1. Structure and Composition

**Figure 1:**
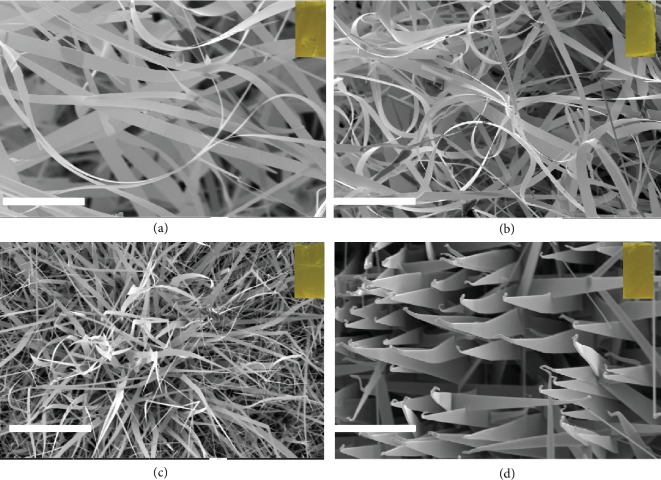
Typical FESEM images of different X (X = F, Cl, Br, or I)-based Ni(II) ions doped CdS nanostructures: (a) NiF_2_-doped CdS nanobelts (sample A), (b) NiCl_2_-doped CdS nanobelts (sample B), NiBr_2_-doped CdS nanobelts (sample C), and (d) NiI_2_-doped CdS nanobelts (sample D) (scale bar = 5 *μ*m). Insets are the optical images of the as-grown samples.

Figures 1(a)–1(d) show the high-magnified FESEM micrographs of CdS:Ni(II)X NBs by samples A-D, representing the NiF_2_-, NiCl_2_-, NiBr_2_-, and NiI_2_-doped CdS samples by CVD growth in the tube furnace. The morphology of doped samples seemed to be affected by the initial precursors [34] but no significant changes. Au nanoparticles were often seen as a catalyst on the top of all the belts, confirming their early vapor–liquid–solid (VLS) growth mechanism and then some VS growth to the belt. A similar growth mechanism and optical luminescence in NiCl_2_-doped CdS nanoribbons have been studied in a previous report [32].

**Figure 2:**
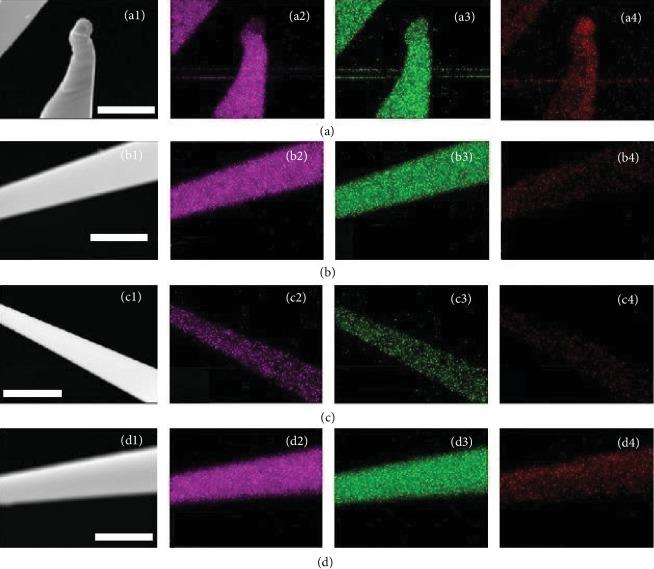
(a–d) EDX mappings of NBs doped with NiF_2_, NiCl_2_, NiBr_2_, and NiI_2_, showing the uniform spatial distribution of Cd, S, and Ni elements (scale bar = 500 nm).

All the samples (A, B, C, and D) were investigated with FESEM-EDX which confirmed that NBs are composed of dominant elements Cd, S, and Ni; sample A-D EDX spectra are displayed in [Supplementary-material supplementary-material-1], in which there is no F and Cl elements found in the samples A and B, while the Br and I elements have been detected in samples C and D because of their larger atomic number in the gas-phase growth. Furthermore, their elemental distribution was probed by EDX mapping (Figures 2(a)–2(d)) for the Cd, S, and Ni elements. Cd, S, and Si are dominant; minor Ni (854 eV), I (618 eV and 386 eV), and O (530 eV) elements can also be detected. The Si element comes from the substrate; O should come from the surface-adsorbed O_2_ and O element in the surface of silicon wafer.

The crystallinity of the doped NBs was analyzed by high-resolution TEM (HRTEM). [Supplementary-material supplementary-material-1] shows the HRTEM images of sample A to sample D, respectively, in which there are atomic separation fluctuations of 0.358, 0.357, and 0.359 nm, respectively, which is the atomic spacing value change of (100) plane for the hexagonal CdS structure. The slight variation of the “d” value might be due to different atomic doping of Ni^2+^ and X^−^ into the CdS lattice. In the HRTEM image, we can also see the varied contrast distribution and sparsely cloudy atom order, which is different from the clean order for the pure crystal. That cloudy stuff usually comes from the dopants in CdS lattice. Insets are the corresponding selected area electron diffraction (SAED) patterns confirming that the NBs are a single crystalline CdS wurtzite structure.

X-ray diffraction (XRD) patterns of four differently CdS:Ni NBs are shown in [Supplementary-material supplementary-material-1]. In all the cases, the prominent peaks at 24.2, 26.4, 28.2, and 47.7° are originated from the (100), (002), (101), and (110) planes of the wurtzite CdS structure (JCPDS card no. 65-3414), and the XRD profiles of (100), (002), and (101) peaks are the same for the NiCl_2_-, NiBr_2_-, and NiI_2_-doped CdS NBs with the strongest (101) peak, while the NiF_2_-doped CdS NBs have a different profile with its strongest (002) peak. No peaks related to any other lattice phases of CdS and NiX_2_ were found, matching with their EDX results. For comparison, the X-ray diffraction pattern of pure CdS NBs is presented in [Supplementary-material supplementary-material-1], whose profile is much like that of Ni(Cl, Br, I)_2_-doped NBs. All the diffraction patterns were fitted with the multipeak Gaussian function (insets of [Supplementary-material supplementary-material-1]) and obtained FWHM along with precise peak positions. All the peak shifts and FWHM are listed in [Supplementary-material supplementary-material-1], in which there is a small shift for the doped belts, as compared to pure NBs. This shift may be due to the lattice strain caused by the incorporation of doping atoms.

CdS crystallize in a hexagonal wurtzite structure which belongs to the *P6*_3_mc-C^4^_6v_ space group, and it has nine optical phonon modes along with three acoustic phonon modes [35]. [Supplementary-material supplementary-material-1] shows the Raman spectra of four different samples and pure CdS NB ([Supplementary-material supplementary-material-1] Raman spectra of pure CdS NB) measured in the spectral range 180-750 cm^−1^ at room temperature by 532 nm excitation. In CdS crystal, the macroscopic electric field associated with longitudinal optical (LO) vibrations make the LO mode energy greater than the transverse optical (TO) mode energy. This effect removes triple degeneration in the Brillouin zone center, producing double degeneration of LO mode and single degeneration of TO mode. The identity of these modes is well established by their polarization characteristics as described in Ref. [35]. On the basis of the above-mentioned group theory explanation, the observed five modes are attributed to 1TO, E_1_(TO), E_2_^H^, 1LO, and 2LO phonon modes of CdS [36, 37]. All the modes are listed in [Supplementary-material supplementary-material-1]; most of the modes redshifted as compared to respective modes of pure CdS NB, confirming the successful incorporation of Ni ions or halogen ions into CdS lattice [38].

### 2.2. Magnetic Properties

**Figure 3:**
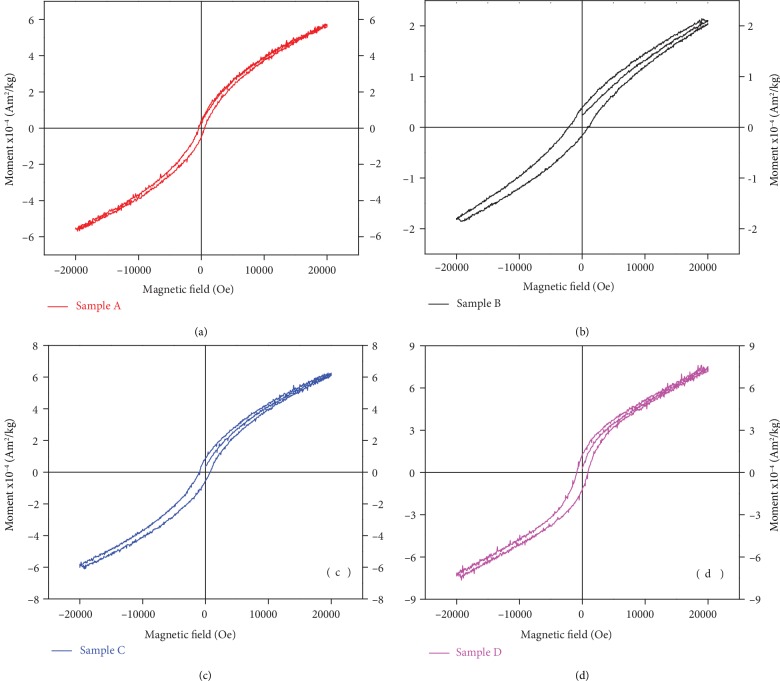
(a–d) Room temperature *M*‐*H* plots of samples A, B, C, and D, respectively.

The magnetic properties are investigated by measuring M-H curves in the range -20000 to 20000 Oe using VSM. Figures 3(a)–3(d) represent the magnetic response of samples A, B, C, and D, respectively, at room temperature. Magnetic hysteresis loop of Si substrate and pure CdS NBs demonstrate diamagnetic properties (not shown here). After doping with NiX (X: Cl_2_, F_2_, Br_2_, and I_2_), the NBs demonstrate ferromagnetism. The *M*‐*H* curves show good magnetic hysteresis loops, clearly indicating the ferromagnetic behaviour. Their saturation magnetization *M*_s_, remanence *M*_r_, and coercivity *H*_c_ can be roughly obtained in [Supplementary-material supplementary-material-1] for the perpendicular detection. It can be seen from [Supplementary-material supplementary-material-1]; sample D show the strongest magnetic signals among the four samples while the weakest magnetic signals occur in the NiF_2_-doped CdS NBs. The anisotropic magnetic effect [39] and Ni-Ni coupling [32] in NiX cluster should be the cause of its magnetism. These results indicate that these CdS NBs doped by NiX_2_ are diluted magnetic semiconductors.

In a dilute magnetic semiconductor, minor doping often produces inhomogeneous doping ion distribution; both single TM ion and coupled TM ions contribute to the magnetic responses in wide-band II-VI DMS [40]. So, the accurate determination for the magnetic parameters is hard to realize.

### 2.3. Optical Properties

**Figure 4:**
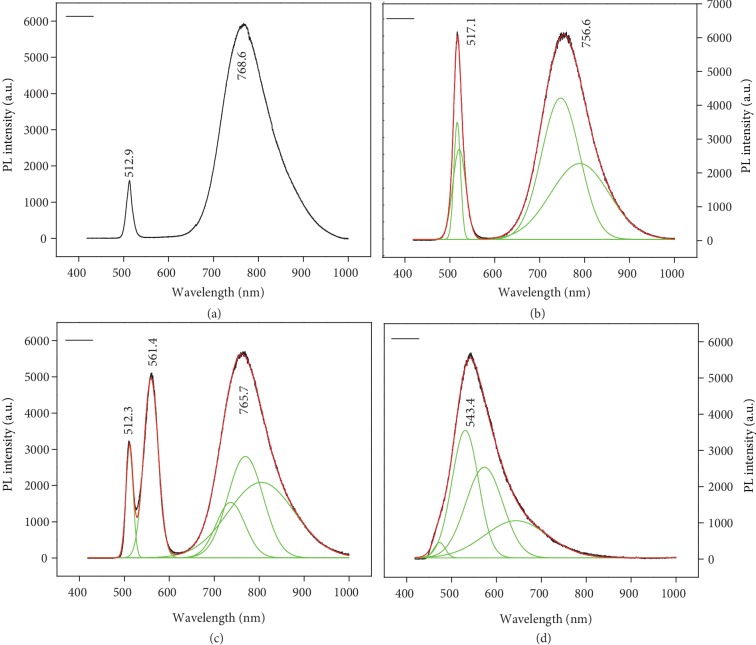
(a–d) PL spectra of samples A, B, C, and D, respectively. All the samples are excited with a CW laser of wavelength 405 nm.

For the photoluminescence (PL) study, Figures 4(a)–4(d) show the PL spectra of all the samples (A, B, C, and D) under the 405 nm CW laser. All the PL spectra are recorded in the range 450 to 1000 nm. [Supplementary-material supplementary-material-1] shows the PL of pure CdS NB, whose narrow emission band centered at about 508-510 nm is attributed to the bandedge (BE) emission from the free exciton recombination of CdS crystal. There is no broadband detected in the longer wavelength range, confirming free of Cd or S vacancies under such growth condition [41, 42]. Figures 4(a)–4(c) show PL spectra for the individual NBs of sample A, sample B, and sample C, respectively. Samples A and B show only two emission bands, one lying at near bandedge (NBE) and the other lies at the near infrared (NIR) region. The former comes from FX+EMP, while the latter should come from d-d transition of Ni ion and (NiX)_*n*_ (X = S or F and Cl, *n* ≥ 2) [32, 33, 43]. In contrast, sample C shows three emission bands, two bands lying at NBE region and one at NIR region. In all three A, B, and C samples, their first peaks all slightly redshifted as compared to that of the pure CdS NB as shown in [Supplementary-material supplementary-material-1]. This slight redshift indicates that the first emission band should contain the contribution of exciton magnetic polaron (EMP) [7, 30]. The emission band in the range from NIR of 700–850 nm in all three samples is attributed to d-d transitions of Ni ion or (NiX)_*n*_ aggregates [32], whose d levels have minor dependence on the weak crystal field effect of Ni ion [44], but the covalence effect takes effect in the order of NiI_2_ > NiBr_2_ > NiCl_2_ > NiF_2_. In samples A and B, the dominant d-d transition bands are located at 768 nm and 756 nm for CdS:NiF and CdS:NiCl, respectively [30]. In sample C, the bands similar to the CdS:NiF NB have been detected; moreover, one new emission band appeared at 561.4 nm. This emission band at 561.4 nm is partially attributed to the high d-d level transition between ^3^A_2_g and ^1^E_g_ of Ni ions in bromides [45] or the self-trapped exciton due to Br ions in CdS lattice [46]. The latter assignment should be more favourable here, which will be discussed later.

Figure 4(d) shows the PL spectrum of individual NBs of sample D which is obtained from the chemical vapor deposition of CdS with NiI_2_ in the gas phase. Although these belts contain dopant ions as confirmed by EDX ([Supplementary-material supplementary-material-1]), XRD ([Supplementary-material supplementary-material-1]), and Raman analysis ([Supplementary-material supplementary-material-1]), they show completely different emission spectra from those of samples A, B, and C. First, there is a very large redshift and even broader emission band centered at 543.4 nm than those for other Ni compound-doped NBs, far from the bandedge emission at 506 nm for pure CdS. Second, there is no prominent emission band detected in NIR region as that in Figures 4(a)–4(c). Extending and comparing with the emission profile of CdS:NiBr NB, we did careful fitting on the broad emission band of CdS:NiI and obtained the separated bands at 473 nm, 530 nm, 572 nm, 644 nm, and 773 nm. The first one at 473 nm may be the partial existence of bound magnetic polaron (BMP) pair due to the free exciton coupling with antiferromagnetic-coupled Ni ions in CdS lattice [47] that can only happen at more p-d hybridization and covalence in iodides [44]. The 530 nm band should be the EMP, that is, FX coupled with ferromagnetically coupled Ni(II) clusters [7] in CdS lattice, which should be the most stable excitation near the bandedge of CdS:NiI NB for the low doping level. The 573 nm band should be the self-trapped exciton [46] due to I^−^ ion in CdS lattice. Its assignment is reasonable here that the self-trapped exciton component by independent I^−^ ion should have more contribution and a lower energy than Br^−^ in NiBr_2_ doping system because I^−^ could be more easily reduced and easily deposited than Br^−^ in CdS lattice. The broadband at about 644 nm should contain the d-d transitions of Ni ions [32, 33, 43] and their aggregates due to its very broad width. We also found a 773 nm band but has a very low ratio, which should be due to the magnetic polaron pair. Our assignments were also supported by the EDX result as shown in [Supplementary-material supplementary-material-1]; the Ni concentration (at 854 eV) is higher than I (at 618 eV), though adsorbed O 1s were also detected. In [Supplementary-material supplementary-material-1], the EDX spectrum of CdS:NiBr contains Ni and Br elements, but the spectra of CdS:NiCl and CdS:NiF contain no Cl and F elements, respectively. These results prove that the existence of Br and I in the lattice has relations to the self-trapped exciton in CdS:NiX NBs. Hence, the above assignment of the very broad emission band around 543 nm in CdS:NiI_2_ NB, which is far from the bandedge (508 nm) of CdS crystal and the d-d transition (650-700 nm) of Ni ions, should contain some mobile and wide-band, covering the range of 450-800 nm due to Ni-I charge-transfer. This band can combine with the conduction band (above 500 nm) of CdS, EMP, self-trapped exciton, and the d-d transition of Ni ions. Such a combined state may participate in the emission of CdS:NiI, but not all the above individual states contribute to radiative transition as in CdS:NiF, CdS:NiCl, and CdS:NiBr. [Supplementary-material supplementary-material-1] present all the reflection spectra of different compound-doped CdS NBs; only the NiI_2_-doped NBs show significant absorption in the range of 510-650 nm, which is in agreement with the above-combined state, especially the Ni-I charge-transfer band. The occurrence of the combination state may reflect another electronic profile with those diluted magnetic semiconductor without doping negative ions like I or Br, i.e., the charge-transfer band between dopant ions like Ni-I may involve the combination via p-d hybridization, which bring up the formation of magnetic polarons and polaron-polaron interaction [47]. Therefore, 473 nm and 530 nm bands are the antiparallel and parallel-coupled spin states out of Ni ions coupling to the free exciton in CdS lattice, respectively.

**Figure 5:**
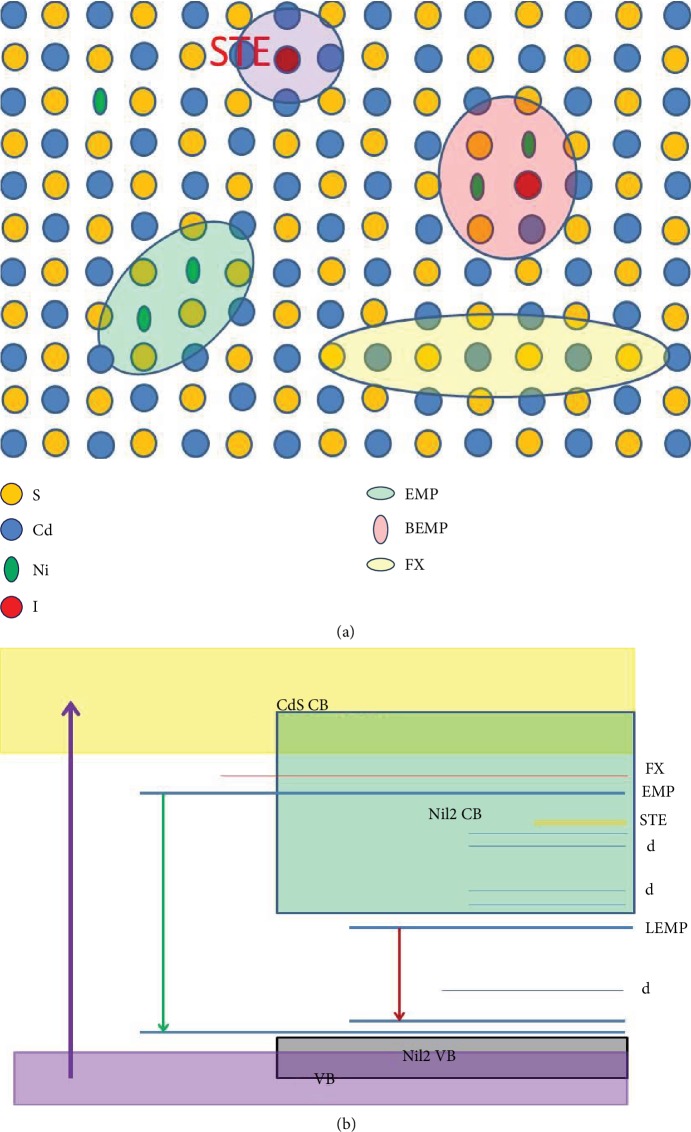
(a) The spatial distribution diagram of the different excited states in NiI-doped CdS lattice. (b) The electronic structure of CdS:NiI_2_ NB, in which the nanocluster of NiI_2_ is incorporated.

From the doping situation, the band structures of NiI_2_-doped CdS structure could be calculated. Except self-trapped exciton, other states could be seen in their band structure as shown in [Supplementary-material supplementary-material-1] by VASP ab initio calculation. If I element is codoped within the CdS lattice, the charge-transfer bandedge of NiI cluster could merge with the up d state above the Fermi level, which may take part in the optical d-d transition. If I ion is not doped into lattice, this cannot happen. In fact, we found that both Br and I could be easily left into the CdS lattice from their micro-photoluminescence spectra in Figure 4. As the phenomena described above, we can figure out the Ni and X doping spatial diagram (a) in DMS CdS lattice and the band or level profile (b) of different states as shown in Figures 5(a) and 5(b). The single Ni, I, coupled Ni, and coupled Ni+I doping states coexisted in the lattice (Figure 5(a)), forming an inhomogeneous DMS structure. They can form different levels within the bandgap out of their different interactions in CdS lattice (Figure 5(b)). These levels may lead to novel optical processes, especially the one coupled to the d state.

### 2.4. Lasing Properties

To know this unusual emission behaviour of sample D as compared to samples A, B, and C, we excited their single NBs with a femtosecond laser pulse of 405 nm. Surprisingly, samples A, B, and C did show the lasing line near CdS bandedge only at very low dopant concentration as usual [7], but no other lasing lines as shown in [Supplementary-material supplementary-material-1]. The lasing profiles of CdS:NiF_2_, CdS:NiCl_2_, and CdS:NiBr_2_ nanobelts in [Supplementary-material supplementary-material-1] show similar lasing profile with varied threshold powers. For CdS:NiF_2_ NBs, sometimes, it may show double lines near the bandedge (514-525 nm) at initial excitation due to overdoping (Ni > 5%) and varied magnetic phases, but at higher power, one mode lasing line still dominated. We observed that only CdS:NiI_2_ belts showed dual lasing lines with large span at high power in different transition zones out of its unique character.

**Figure 6:**
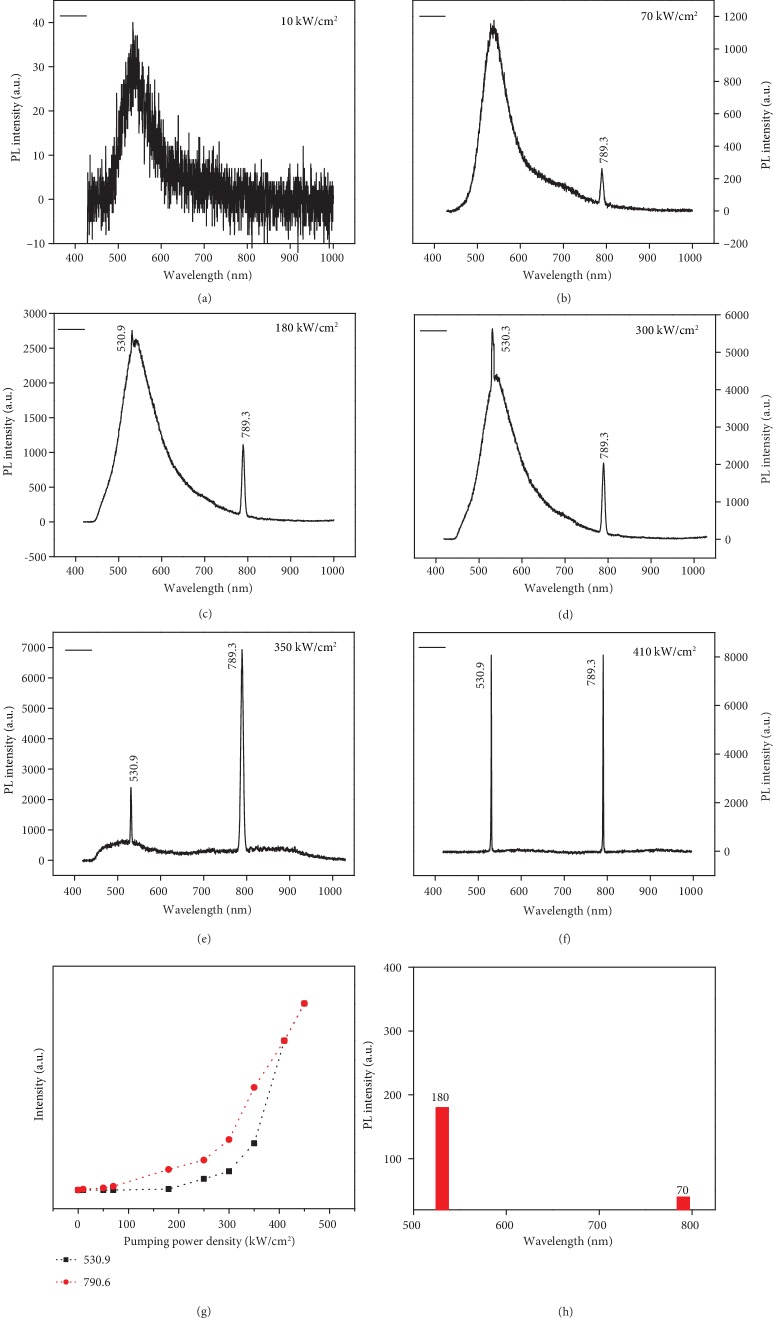
The power-dependent emission spectra at 10 kW/cm^2^ (a), 70 kW/cm^2^ (b), 180 kW/cm^2^ (c), 300 kW/cm^2^ (d), 350 kW/cm^2^ (e), and 410 kW/cm^2^ (f) of single CdS:NiI NB; its power vs. PL intensities (g) and threshold power and wavelength (h).

High doped (over 1% Ni) CdS NB often could not produce the lasing line at all. However, sample D NBs showed a variation of both spectral and lasing profiles as shown in Figures 6(a)–6(d). For low excitation fluence (10 kW/cm^2^), NB showed the only broad emission band centered at 534.5 nm (EMP at 530 nm+self-trapped exciton at 572 nm) and weak band-tail from 630 to 800 nm (d-d transition). But when increasing the excitation fluence (≥70 kW/cm^2^), its spectra are shown in Figure 6(b), in which an extremely very narrow lasing line (width 1.5 nm) appears at 789.3 nm, but the green emission band at 535 nm did not give a narrow line though its intensity increases. Further increasing the pump fluence from 70 to 180 kW/cm^2^, the narrow line at 789.3 nm rises, and another very narrow line at 530.9 nm appears, which is shown in Figure 6(c). Interestingly, with further increasing pump fluence up to 300 kW/cm^2^, the line at 530.9 nm increases much faster than the line at 789.3 nm, which was shown in Figure 6(d). After the pump fluence over 350 kW/cm^2^, the line at 789.3 nm increases faster than the other one as shown in Figure 6(e). When the pump fluence goes up to 400 kW/cm^2^, both sharp lasing lines increase superlinearly further without saturation as shown in Figure 6(f). Figure 6(g) shows the excitation fluence-dependent intensities for lines at 530.9 nm and 789.3 nm, respectively. Lasing thresholds for lines 789.3 nm and 530.9 nm are seen to be 70 and 180 kW/cm^2^, respectively, as shown in Figure 6(h). This fact proves that the Ni and I element incorporations in the lattice of CdS NB play an important role in their dual-color lasing profile.

**Figure 7:**
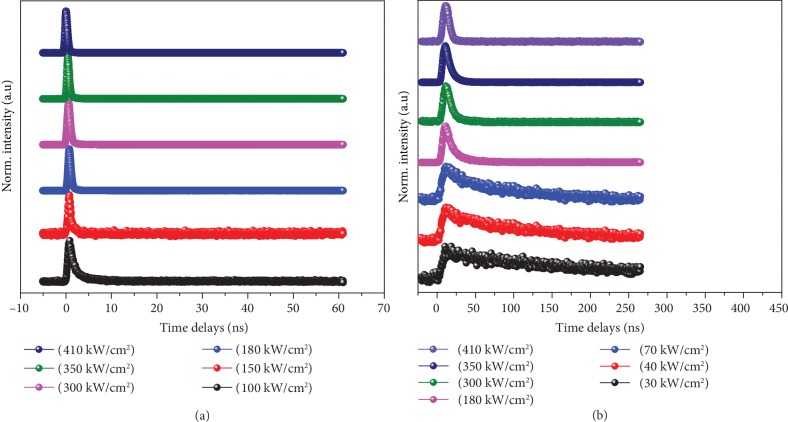
The PL lifetime decay profiles of the emission at (a) 530.9 and (b) 789. 3 nm with different pump fluences.

Lasing characteristics are further confirmed by their PL lifetime measurements. Figures 7(a) and 7(b) show the decay curves for 530.9 and 789.3 nm emissions of CdS:NiI_2_ NB at the pump fluences of 180, 300, 350, and 410 kW/cm^2^, respectively. Their single-exponential decay function and biexponential decay function were used to fit the PL decay curves presented in Figures 7(a) and 7(b), respectively. Their lifetime variations with excitation powers were listed in [Supplementary-material supplementary-material-1]. For 530.9 nm emission, the first-lifetime component decreases from 0.95 ns to 0.28 ns as the laser fluence increases from 100 to 410 kw/cm^2^, whose ratio increases from 89% to 98% at initial, then decreases to 72%. The second lifetime component does the same thing from 3.72 ns to 1.12 ns. Their lifetime decreasing and intensity superlinear increasing indicate a stimulated emission process with the rising excitation power, in which the EMPs were produced at first and then they were converted into a coherent state—collective EMPs (CEMP) at high excitation [7, 48]. Over a critical power, the ratio of second lifetime component decreases with rising power reflecting the occurrence of some competition processes or scattering effect, which could be identified by the threshold value for lasing in Figure 6(g).

For the 789 nm emission, the first component lifetime increases at powers below its threshold value (70 kW/cm^2^), while the second component shows a tendency towards lifetime reduction as shown in Figure 7 and [Supplementary-material supplementary-material-1], combining with their superlinear intensity rising. The second component always has the dominant ratio. This emission process presents a clear coherent radiation process. Compared with the EMP behaviour in CdS:Mn [30] and CdS:Co NB [7], the emission bands at 530.9 nm and 789 nm in CdS:NiI_2_ NB have clear lifetime profiles of the EMP and LEMP, respectively; both of them can convert into CEMP for coherent emission. Their lifetime variations hint that these EMPs have even larger stabilities and faster populations than other states, stronger emission coherence, which supply more probabilities for CEMP formation, and related coherent radiation transition. Two CEMP cooccur in the same location in DMS. What does that indicate?

## 3. Discussion

The photoluminescence of NiX_2_ compounds comes from their absorption if it could occur. NiF_2_ [49], NiCl_2_, NiBr_2_ [33], and NiI_2_ [43] crystals show very similar absorption profiles due to their Ni(II) d-d transition in the range of 600-800 nm, over which there is an extended charge-transfer (CT) absorption bandedge for only NiI_2_ shifted to 900 nm, superposed with the d-d transition bands. The gas-phase absorption spectra of NiCl_2_ and NiBr_2_ also show similar absorption profile in this 600-800 nm range while the NiI_2_ have no strong absorption in this zone but even lower energy zone [49]. So, the absorption band in 600-720 nm range for bulk NiI_2_ should come from the blueshift of single NiI_2_ molecule band in antiferromagnetic cluster or crystal due to electronic correlation [39, 50]. However, the ferromagnetic nanocluster may lead to their d-d band redshift again. Hence, the 789 nm lasing line should come from the (NiI_2_)_*n*_ ferromagnetic cluster but not single NiI molecule, at which the d-d band or LEMP form [32] near CT bandedge for I ions involved, in the CdS NB lattice. That is to say, the collective LEMP could occur near the bandedge of (NiI)_*n*_-doped CdS NB at high excitation as shown in Figure 5(b), but those NiF_2_-, NiCl_2_-, and NiBr_2_-doped CdS NBs have no such state within the bandgap of CdS because the charge-transfer band of Ni-F, Ni-Cl, and Ni-Br has large energy separation to the Ni d state.

Another key factor within a NB in designing nanostructure-based lasers is the quality factor (*Q*) for its active waveguiding, which reflects the optical confinement and amplification of guided modes which couple with the absorption band or levels. For the broad emission band out of a semiconductor NB, the optical cavity effect in such NB can induce multimode lasing with increasing excitation powers [50]. However, in fact, for these CdS:NiCl_2_, CdS:NiBr_2_, and CdS:NiI_2_ NBs, no multimode lasing was seen at all, but only single mode is observed near the CdS bandedge out of EMPs. For CdS:NiF_2_ NB, we can see single-mode lasing or two-mode lasing near its CdS bandedge, whose double lasing lines may come from the different Ni ion aggregates of varied aggregation numbers because scarcely any F were left in the CdS lattice. So, the above observed lasing lines near CdS bandedge did not come from the optical cavity effect in such NB but an exciton scattering from a dense excitonic state [1]. In a diluted magnetic semiconductor NB, only the EMP can contribute to such state and give single-mode lasing favourably [7].

Only the DMS CdS:NiI NB can produce two lasing lines; the span between two lines was large to even 0.75 eV. From the NB size and broad emission band, this NB could form optical microcavity to produce multimodes [25]; however, we can only see one lasing line at 530.9 nm and another lasing line at 789.4 nm with no contribution from the optical cavity. So, we need to think about the other possible origins for such lasing lines in CdS:NiI_2_. Usually, the lasing profile comes from the exciton scattering by the dense exciton near a bandedge of the semiconductor structure with a very low bound carrier density [51] and the CEMP [7] in diluted magnetic semiconductors, but their LEMP (out of d-d transition) never produce lasing lines for local nature so far [30, 32]. The 530 nm and 789 nm lines are very close to the EMP and LEMP levels below the bandedges of CdS crystal and NiI_2_ nanocluster, respectively. Why does lasing lines at 530.9 nm and 789 nm simultaneously show up, especially the latter?

For the CdS wurtzite structure, its bandgap of 2.25 eV (about 500 nm) comes from the charge-transfer band, whose exciton emission and the lasing line can occur near bandedge (505-510 nm). But transition metal compounds (TMC) have strong electronic correlation [50]; the bandgap is usually large due to correlation in the antiferromagnetic phase, and their in-gap d states are in correlation effect and with strong hopping quenching between TM ions. That is to say, TMC bulk cannot emit light like a direct band semiconductor. The d-d radiative transition of TM ion can be allowed in its doping in semiconductors due to p-d hybridization, whose transition energy has relation to its crystal field and spin-spin coupling [52]. Hence, it deserves study why TM ion-doped semiconductor can produce lasing.

The optical band structures of nickel compound are complicated due to the strong electron correlation [50, 53]. Due to the competition of correlation energy *U*, CT energy *Δ* between Ni and I, as well as the electron-phonon interaction in these TMC [50], their band gaps (*E*_g_) reduce significantly from NiF_2_, NiCl_2_, and NiBr_2_ to NiI_2_. The color of NiF_2_, NiCl_2_, and NiBr_2_ powders is light green, light green, and yellow-green, originating from the d-d transition of Ni(II), and their bandgap values are all determined by the strong *U* and exhibited in the ultraviolet and visible zone. But for the NiI_2_ crystal of black color, bandgap should be determined by the CT energy *Δ* with very small value, an infrared absorption band as indicated in Ref. [42]. Its Raman scattering modes by visible laser excitation all screened by the carrier state in CT band could hardly be detected. That is to say, NiI_2_ could overcome the correlation energy *U* to become a covalent compound and narrow bandgap semiconductor or metallic solid under pressure [54]. Of course, it can increase bandgap with reduction of size.

When the NiX_2_ dope into CdS lattice, the F and Cl atoms may evaporate out of the crystal; only Ni ions were left in the lattice as dopants, then Ni ions can aggregate within CdS NB, leading to ferromagnetic response and LEMP emission due to the ferromagnetic coupling between Ni ions [32]. This indicates a ferromagnetic coupling for TM ions in Ni^2+^_*n*_ or (NiI)_*n*_ nanocluster in CdS lattice, but the antiferromagnetic coupling between ferromagnetic layers in NiI_2_ crystal occurs at low temperature [39] but do not show up in minor doping case. Hence, this CdS:NiI_2_ DMS nanostructures could produce EMP emission band as the second band at 530 nm we fitted in Figure 4(d). Moreover, the EMP condensate could form at below CdS bandedge by pulsed laser excitation, which could be identified by a single-mode lasing line close to its EMP energy [7, 48]. That should be why we see the 530.9 nm lasing line in CdS:NiI_2_ NB, and its similar EMP lasing line also was seen in CdS NBs doped with NiF_2_, NiCl_2_, and NiBr_2_ as shown in [Supplementary-material supplementary-material-1]. Why do this EMP line show up so far away from the bandedge of CdS? Two factors are leading: (1) More Ni ions (close to 4%) doped than that in previous works, so their large ferromagnetic coupling would bind more LO phonons to lead to a large redshift. (2) In the previous report, we use Ni, Mn, and Co chloride as precursors for CdS doping. During the growth of CdS NBs, F or Cl ions can be removed by carrier H_2_ gas; hence, they cannot be left in the lattice during growth. So the spin-coupled Ni ions play a dominant role in the emission process of CdS:NiCl_2_ and CdS:NiF_2_. But for NiBr_2_ or NiI_2_ as precursors, I and Br are heavy atoms, hard to be evaporated; hence, they might be left into CdS lattice during growth. Therefore, Br and I ions could also work as codopants with Ni ions in CdS NB. The Br or I ion in the CdS lattice could lead to the charge-transfer effect and the formation of exciton complexes or acceptor-bound exciton or self-trapped exciton (STE) [46], hence bring about a larger redshift of emission band as shown in the STE at 561 nm in Figure 4(c) and the composite band at 543 nm in Figure 4(d). For CdS NBs doped with Ni ions and only minor F, Cl, and Br ions under fs pulse excitation, we can detect their EMP lasing line. For CdS doped with NiF_2_, its lasing lines occur at 513.3 nm (bound magnetic exciton) and 518.4 nm (EMP) as shown in [Supplementary-material supplementary-material-1]; the lasing line for CdS NB doped with NiCl_2_ is at 514.8 nm as shown in [Supplementary-material supplementary-material-1], while the lasing line of the CdS NB doped with NiBr_2_ is at 515.1 nm as shown in [Supplementary-material supplementary-material-1]. A gradual shift of EMP line goes to lower energy from F or Cl to Br or I, for EMP often contain LO phonon coupling [7, 48] and TM ion aggregation number. However, the NBs doped with NiF, NiCl, and NiBr cannot produce NIR lasing line; moreover, slightly higher doping in CdS NB even cannot produce lasing at all wavelengths for efficient carrier scattering. The lasing conditions in the above NBs reflected their exciton nature in DMS. The anomalous lasing behaviour of CdS:NiI relates its low energy PL to the microscopic *Δ* value for (NiI)_*n*_ clusters. Therefore, the LEMP near the charge-transfer bandedge of (NiI)_*n*_ cluster can lead to exciton-like excitations and produce new lasing line in the NIR region [50, 54]. Only the bosonic excitations could coexist in the same real space in a semiconductor and emit light simultaneously; moreover, coupled spins could bind more bosonic excitations via exchange interactions to form a macroscopic quantum state for coherent radiation.

CdS:NiBr_2_ NB has emission bands at 512.3 nm and 561.4 nm as shown in Figure 4(c), but its lasing line is at 515.1 nm from the EMP condensate. So, the 561 nm band should be the STE band due to Br ion incorporation. [Supplementary-material supplementary-material-1] presented the Ni and Br concentration-dependent PL spectra of CdS:NiBr NBs, from which we can separate the emission peaks from FX, EMP, and STE in different wavelengths below CdS bandedge. The STE band occurs or increases with Br concentration, in the range of 550-600 nm. The broadband at 765 nm should be the LEMP as that in NiCl_2_-doped CdS NB [32], originating from the d-d transition of Ni(II) and Ni(II) aggregates; such LEMP with no charge-transfer band involvement cannot produce lasing at all.

CdS NB doped only by NiI_2_ show similar behaviour in its low doping concentration range as shown in [Supplementary-material supplementary-material-1], in which the EMP and LEMP out of Ni_*x*_ cluster could be identified clearly. But for high Ni concentration up to 2-4%, strong bandedge emission was seen out of NiI_2_ cluster in CdS as shown in Figure 4(d). Moreover, it can produce dual lasing lines with giant energy span because of I ion involvement. This supplied a coherent space for the LEMP and an energy band for LEMP, but not a local transition. Ab initio calculation by VASP software presented the band structures of CdS:Ni and CdS:NiI, respectively, as shown in [Supplementary-material supplementary-material-1] based upon the unit of Ni_2_Cd_34_S_36_ and Ni_2_Cd_34_S_35_I. It is found that the FM coupling Ni(II) pair has stability energy lower of 121 meV than that of the AFM pair, so the CdS:Ni DMS form easily due to FM-coupled Ni pairs. If only Ni ions are considered in the lattice, FM coupling between Ni ions modifies their band structures as shown in Figures [Supplementary-material supplementary-material-1]; most of d states are above the Fermi level, which has a small separation to the conduction band minima as shown in [Supplementary-material supplementary-material-1]; moreover, the d state has strong hybridization with the valence bandedge as shown in [Supplementary-material supplementary-material-1]. In such a system, the optical band-band transition would dominate, and the exciton effect plays an important role with FM coupling. But for AFM doping system, the d state distributes cross the Fermi level; the metallic state with d state may dominate as shown in [Supplementary-material supplementary-material-1], so its optical transition would relax via both conduction bandedge and lowest d-d transition independently. For the system with Ni(II) and I codoping in CdS lattice, the energy band structure varied much. For the spin-up state, Ni ion takes minor effect on the band structure as shown in [Supplementary-material supplementary-material-1], while the spin-down state shows a contact between the continuum conduction band and d state; this indicates that the charge transfer between Ni and I could extend its bandedge to lower energy and reach the d state. Even stronger p-d hybridization and continuum Ni-I CT band would combine with Ni FM coupling to lead to the second band minima near the highest d level as shown in [Supplementary-material supplementary-material-1], which could be called I^−^ bound EMP (BEMP), from the overlapping between FM coupled d-d transitions (LEMP) and Ni-I CT band around 700-850 nm. Due to the minor amount of I ion, the continuum band out of Cd-S and sparsely Ni-I miniband would coexist; the latter gets so low to go near cross the highest d state, then inhomogeneous EMP nearby S zone and BEMP around I^−^ zone may show up simultaneously. Based on the above discussion, we can draw a diagram on the exciton spatial distribution in the NiI-doped CdS lattice as shown in Figure 5(a), in which we can see the locations of free exciton, EMP (LEMP), STE, and bound EMP (BEMP). Then, we know that the CdS:NiI_2_ NB has two bandedges out of CdS crystal and NiI_2_ nanocluster; both types of EMPs below their bandedges could form due to aggregated FM Ni ions after photoexcitation above *E*_gCdS_. Both two EMP states can lead to CEMP for lasing at high excitations. Therefore, an electronic state-level diagram of CdS:NiI NB could be drawn in Figure 5(b), in which there occurs a partially mobile bandedge extended to 700-850 nm; in contrast to that, the mobile bandedge of Ni-Cl, Ni-F, and Ni-Br is moved to above the CdS bandedge as shown in [Supplementary-material supplementary-material-1].

For 789 nm lasing line in CdS:NiI NB, energy is lower than the d-d transition (750-760 nm) of single Ni ion in CdS lattice for its EMP nature contains exciton-phonon coupling [7, 32]. From this point, no doubt, it has relation to the d-d transition. However, it is well known that the d-d transition within the bandgap of TMC or DMS can be seen as a local exciton, with usually forbidden selection. It relaxes via some p-d hybridization to lattice and via spin coupling to another TM ion efficiently, so it cannot produce a large number of local excitons enough to form CEMP and emit light coherently. Even high concentration Ni ions doped in a semiconductor, the LEMP decay via efficient hopping mechanism, cannot keep stable and behave coherently. This situation is just the cases in NiF_2_-, NiCl_2_-, and NiBr_2_-doped CdS NB. Though their d-d transitions are important in the solution, it is clear that the d-d transition absorption band cannot be seen in their absorption spectra of the doped NBs (in reflection mode, [Supplementary-material supplementary-material-1]) due to their local nature and low concentration. However, their d-d radiation transition band can be seen easily due to the FM coupling of TM ions and then LEMP formation even for the high d level and/or lowest d level [51]. In spite of LEMP formation, their lasing lines from LEMP cannot occur due to the strong d-d correlation and efficient hopping relaxation in NiF_2_-, NiCl_2_-, and NiBr_2_-doped CdS NB. But for NiI_2_-doped CdS NB, the coupling of local d state (LEMP) and delocalized CT state leads to a merged state, in which FM-coupled Ni ions are surrounded by I and S ions. This merged state is the lowest state above the Fermi level as shown in [Supplementary-material supplementary-material-1] and similar to the mobile covalent band (itinerant state) near the d-d transition region [50]. Therefore, the LEMP within CdS bandgap can form BEMP out of the combination of FM-coupled TM aggregate and CT bandedge of doping M-L cluster in semiconductor lattice, producing a single-mode lasing by fs-laser excitation, together with that the EMP lasing line at 530 nm.

Due to the spatial and energy difference between these two EMPs in Figure 5(b), the BEMP (LEMP) level has a much longer lifetime, lowest energy, and limited space, so it can enter into a CBEMP state at a smaller threshold to produce lasing first after fs pulse pumping. Because natural LEMPs are of the narrow d state of Ni(II) with strong electron correlation, whose population would be easily saturated though combination to the CT band in doped NiI_2_ nanocluster, their high excited d levels or EMP level would start to populate in quantity after CBEMP emit coherently. When the concentration of EMPs reaches their threshold, the EMP start to produce its lasing due to the CEMP formation with coherent emission. No doubt that coexistence of CEMP and CBEMP (CLEMP) in CdS:NiI NB could occur due to their common bosonic nature; moreover, FM-coupled spin cluster favours to bind the multiexcitons via an exchange, producing a EMP condensate for single-mode lasing. Dual lasing lines mean that the EMP and LEMP are robust to give their coherent emissions, respectively. This fact is useful for the study of exciton condensation stabilized by spontaneously coupled spins in a DMS structure and multicolor quantum modulation. This multiline lasing phenomena from EMPs seem to be like the multiexcitons binding by different optical modes in a semiconductor optical cavity [15], but this is realized via spin-spin coupling. The multilevel of macroscopic quantum states in identical semiconductor structure should have grand applications in the future.

It is well known that the strong correlation TM compounds possess rich physical properties due to the tuning of their correlation energy *U*, charge-transfer *Δ*, and electron-phonon coupling S [50]. For example, layered iron pnictides and chalcogenides [55] all are superconductors; the *U* values in these compounds could be modified by the charge-transfer *Δ* and structural effect as indicated by the Zaanen et al. framework [50], leading to a zero-energy bandgap; spin fluctuation and phonon coupling would add to lead to carrier pairing or condensate for superconducting. This superconducting is the case for carrier coherence in the strong correlation system, CT, and spin coupling contributes. If taking into account both size and exciton effects, and putting TMC nanocluster into a semiconductor lattice, (1) the TMC cluster bandgap would blueshift, modifying metallic state (bulk) to semiconducting state (cluster), leading to the exciton state out of the spatial confined CT state in TMC; (2) the spin coupling would be modulated by the size effect; FM coupling dominating with the exciton effect occurs in DMS to produce the LEMP or BEMP and further CBEMP lasing; the latter is a spin-polarized excitonic coherence process. Therefore, the modulation of spin coupling, electronic correlation, and CT in DMSs is important and useful to modify their electronic structures and properties.

In all, we fabricated a series of CdS NBs doped with nickel halide via CVD growth and found that two bandedges coexist in CdS NB doped with NiI_2_; the FM-coupled spin cluster (NiI)_*n*_ could combine with the free exciton to stabilize EMP near the CdS bandedge and LEMP near a d-d transition. The LEMP turn into BEMP if I ion joins the FM Ni pair or cluster, which can produce their independent lasing line, like the EMP near CdS bandedge. This is a new and valuable technique to tune the intrinsic microscopic interactions in strongly correlated compound via size and spin doping in a DMS, to modify their electronic band structure and excitonic nature, producing multi-spin-coupled polaronic states, therefore leading to their new magnetic and spin-related optical properties.

## 4. Materials and Methods

### 4.1. Growth of NiF_2_-, NiCl_2_-, NiBr_2_-, and NiI_2_-Doped CdS NBs

All the chemicals were purchased from Alfa Aesar and are of analytical grade. A high-temperature furnace equipped with a quartz tube was utilized to grow the NBs. For the collection of nanomaterials, *n*-type Si (111) was used as substrates. For this purpose, firstly, Si substrates were sonicated in acetone, isopropanol, and deionized H_2_O for 15 min., respectively, and then dried with pressurized N_2_ flow. After cleaning, a thin layer of Au (~5 nm) was deposited on these substrates with the help of a Quorum (Q150S) sputter coater and loaded into the downstream region of the quartz tube. 1.5 g mixture of pure CdS (99.999%) and X (X: NiF_2_, NiCl_2_, NiBr_2_, and NiI_2_) nanoscale powders was used as a precursor. These precursors were loaded in a ceramic boat and inserted into the middle region of the quartz tube. To achieve the best crystallinity of the nanomaterials, oxygen-free environment was obtained inside the quartz tube with the help of a mechanical and turbo molecular pump. After that, the furnace temperature was raised to 750°C at the rate of 15°C/min and then stayed at 750°C for 25 min. During the reaction, the flow rate of Ar/10%H_2_ carrier gas was maintained to 10 sccm. After 25 min of reaction, a thick yellow-colored product was collected on the Si substrates. The product obtained from CdS:NiF_2_, CdS:NiCl_2_, CdS:NiBr_2_, and CdS:NiI_2_ precursors was named as sample A, sample B, sample C, and sample D, respectively.

### 4.2. Characterizations

Yellow-colored product morphologies were investigated by field emission scanning electron microscopy (FESEM, FEI Quanta 450 FEG) attached with energy-dispersive X-ray spectroscopy (EDX) Thermo (Noran System 7: Ultradry 30 mm^2^) facility. The transmission electron microscopy (TEM) observations were carried out on a Hitachi H-800 microscope with an accelerating voltage of 200 kV. Phase purity of the as-prepared products was investigated by X-ray diffraction (XRD, Bruker: D8-Advance with Cu K*α* radiation of wavelength 1.5418 Å). The 2*θ* ranges from 20 to 60° with a step of 0.01° with a count time of 2 s. Raman experiments were conducted in a Raman scattering spectrometer (LABRAM-HR, JY, Horiba), with the 532 nm Ar laser as the excitation source. Photoluminescence (PL) spectra were obtained by Laser Confocal PL microscopy (Horiba JY-iHR550, Olympus BX51M) using 405 nm continuous wave (CW) semiconductor laser as an excitation source. The laser beam was focused onto the sample by a microscope with a 50x objective lens. PL lasing spectra were measured by using Ti-sapphire laser (coherent, 405 nm, 130 fs, 80 MHz) as a pumping source. The fs-laser beam size was optimized using the lens in front of the microscope to give a circular beam spot diameter of 3 *μ*m to ensure uniform illumination of the doped NBs, and the output signals were collected and analyzed by a spectrometer. PL lifetime decay profiles were recorded by the time-correlated single photon counting (TCSPC) system (PicoQuant “TimeHarp 200”). The pulsed diode laser at 405 nm (tens of ps pulse-width) was used (PicoQuant “PDL 800-B,” 10 MHz-80 MHz) as the excitation source. The measurements of magnetization (*M*) as a function of *H* were characterized in a VSM in the range −2000 ≤ *H* ≤ 2000 Oe.

## 5. Calculation

The band structure calculation shown in [Supplementary-material supplementary-material-1] was carried out by using VASP software via the method indicated by the following references (Kresse G and Joubert D, From ultrasoft pseudopotentials to the projector augmented-wave method *Phys. Rev. B* 59 1758(1999); Blöchl P E, Projector augmented-wave method *Phys. Rev. B* 50 17953(1994); Perdew J P, Burke K and Ernzerhof M 1996 Generalized gradient approximation made simple *Phys. Rev. Lett.* 77 3865(1996)).

## Supplementary Materials

Supplementary Materials Figure S1: FESEM-EDX of samples A (CdS:NiF2), B (CdS:NiCl2), C (CdS:NiBr2), and D (CdS:NiI2), respectively. Figure S2: (a–d) HRTEM image of the selected NBs and its corresponding SAED pattern depicted as insets (scale bar = 5 nm). Figure S3: (a–f) XRD patterns of the samples A, B, C, and D and pure CdS nanobelt, respectively. Insets are the zoomed area from 24 to 30° fitted with multipeak Gaussian function. Table S1: peak shifts and FWHM of major XRD peaks of all the samples (Figure S3). Figure S4: (a–d) Raman spectra of samples A, B, C, and D, respectively. Table S2: list of the Raman frequencies derived from the Raman spectra shown in Figure S4. Table S3: magnetic parameters (saturation magnetization *M*_s_, remanence *M*_r_, and coercivity *H*_c_) of samples A, B, C, and D derived from Figure 3. Table S4: near bandedge (NBE) emission peak energy derived from the PL spectra shown in Figure 4. Figure S5: the PL of pure CdS NB. Figure S6: the absorption spectra of CdS:NiF2, CdS:NiCl2, CdS:NiBr2, and CdS:NiI2 NBs by the reflectance mode. Figure S7: (a and b) the energy DOS structures of CdS:Ni (left) for Ni-Ni cluster ferromagnetic (FM) and antiferromagnetic coupling (AFM) by ab initio calculation; (c) contains two energy band structures for FM Ni(II)-coupled CdS with spin-up states (left) and spin-down state (right); (d) contains two energy band structures for AFM Ni(II)-coupled CdS with spin-up states (left) and spin-down state (right); (e) is the DOS structure of CdS+FM Ni2+I system; (f) shows two energy band structures for FM Ni(II) pair with spin-up (left) and spin-down states. Figure S8: the lasing profiles of CdS:NiF2, CdS:NiCl2, and CdS:NiBr2 nanobelts under fs pulse excitation. Table 5: pump fluence dependent lifetimes for *λ*em: 530.9 and *λ*em: 789.3 nm in sample D. Figure S9: the Ni and Br dopant concentration-dependent PL spectra of CdS:NiBr NBs. Figure S10: the electronic state diagrams of CdS:NiCl2 and CdS:NiBr2 NBs, in which the green band represented the charge-transfer band Ni-X in CdS NBs.
