# Supplementary material for: Dual-Color Lasing Lines from EMPs in Diluted Magnetic Semiconductor CdS:NiI Structure
Source: Research (Wash D C). 2019 Nov 5;2019:6956937. doi: 10.34133/2019/6956937 (PMC6944495; doi:10.34133/2019/6956937)
Supplement: Supplementary Materials — Figure S1: FESEM-EDX of samples A (CdS:NiF2), B (CdS:NiCl2), C (CdS:NiBr2), and D (CdS:NiI2), respectively. Figure S2: (a–d) HRTEM image of the selected NBs and its corresponding SAED pattern depicted as insets (scale bar = 5 nm). Figure S3: (a–f) XRD patterns of the samples A, B, C, and D and pure CdS nanobelt, respectively. Insets are the zoomed area from 24 to 30° fitted with multipeak Gaussian function. Table S1: peak shifts and FWHM of major XRD peaks of all the samples (Figure S3). Figure S4: (a–d) Raman spectra of samples A, B, C, and D, respectively. Table S2: list of the Raman frequencies derived from the Raman spectra shown in Figure S4. Table S3: magnetic parameters (saturation magnetization Ms, remanence Mr, and coercivity Hc) of samples A, B, C, and D derived from Figure 3. Table S4: near bandedge (NBE) emission peak energy derived from the PL spectra shown in Figure 4. Figure S5: the PL of pure CdS NB. Figure S6: the absorption spectra of CdS:NiF2, CdS:NiCl2, CdS:NiBr2, and CdS:NiI2 NBs by the reflectance mode. Figure S7: (a and b) the energy DOS structures of CdS:Ni (left) for Ni-Ni cluster ferromagnetic (FM) and antiferromagnetic coupling (AFM) by ab initio calculation; (c) contains two energy band structures for FM Ni(II)-coupled CdS with spin-up states (left) and spin-down state (right); (d) contains two energy band structures for AFM Ni(II)-coupled CdS with spin-up states (left) and spin-down state (right); (e) is the DOS structure of CdS+FM Ni2+I system; (f) shows two energy band structures for FM Ni(II) pair with spin-up (left) and spin-down states. Figure S8: the lasing profiles of CdS:NiF2, CdS:NiCl2, and CdS:NiBr2 nanobelts under fs pulse excitation. Table 5: pump fluence dependent lifetimes for λem: 530.9 and λem: 789.3 nm in sample D. Figure S9: the Ni and Br dopant concentration-dependent PL spectra of CdS:NiBr NBs. Figure S10: the electronic state diagrams of CdS:NiCl2 and CdS:NiBr2 NBs, in which the green band represented the charge-tran [file 6956937.f1.docx]

**Supporting Information**

**Dual Color Lasing Lines from EMPs in Diluted Magnetic Semiconductor CdS:NiI Structure**

Muhammad Arshad Kamran^1*^, Bingsuo Zou^2,3*^, Kang Zhang^3^, Xiongtao Yang^3^, Fujian Ge^3^, Lijie Shi^3^, Thamer Alharbi^1^

^1^Department of Physics, College of Science, Majmaah University, Al-Majmaah, 11932, Saudi Arabia.

^2^ Key Lab of Featured Metal Resources Utilization and Advanced Materials Development; Nano and Energy Research Center, School of Physics, Guangxi University, Nanning 530004, China.

^3^Beijing Key Laboratory of Nanophotonics & Ultrafine Optoelectronic Systems, Beijing Institute of Technology, Beijing 100081, China.

Email address: zoubs[@gxu.edu.cn](mailto:zbs@bit.edu.cn), [m.kamran@mu.edu.sa](mailto:m.kamran@mu.edu.sa)

**(a)**

**(b)**

**(d)**

**(c)**

**FIGURE S1**(a-d)： FESEM-EDX of samples A (CdS:NiF_2_), B(CdS:NiCl_2_), C (CdS:NiBr_2_), and D (CdS:NiI_2_) respectively.

**(b)**

**(a)**


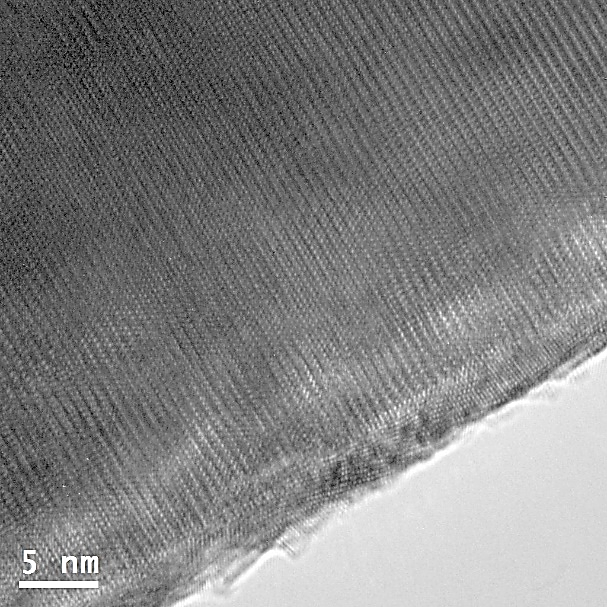

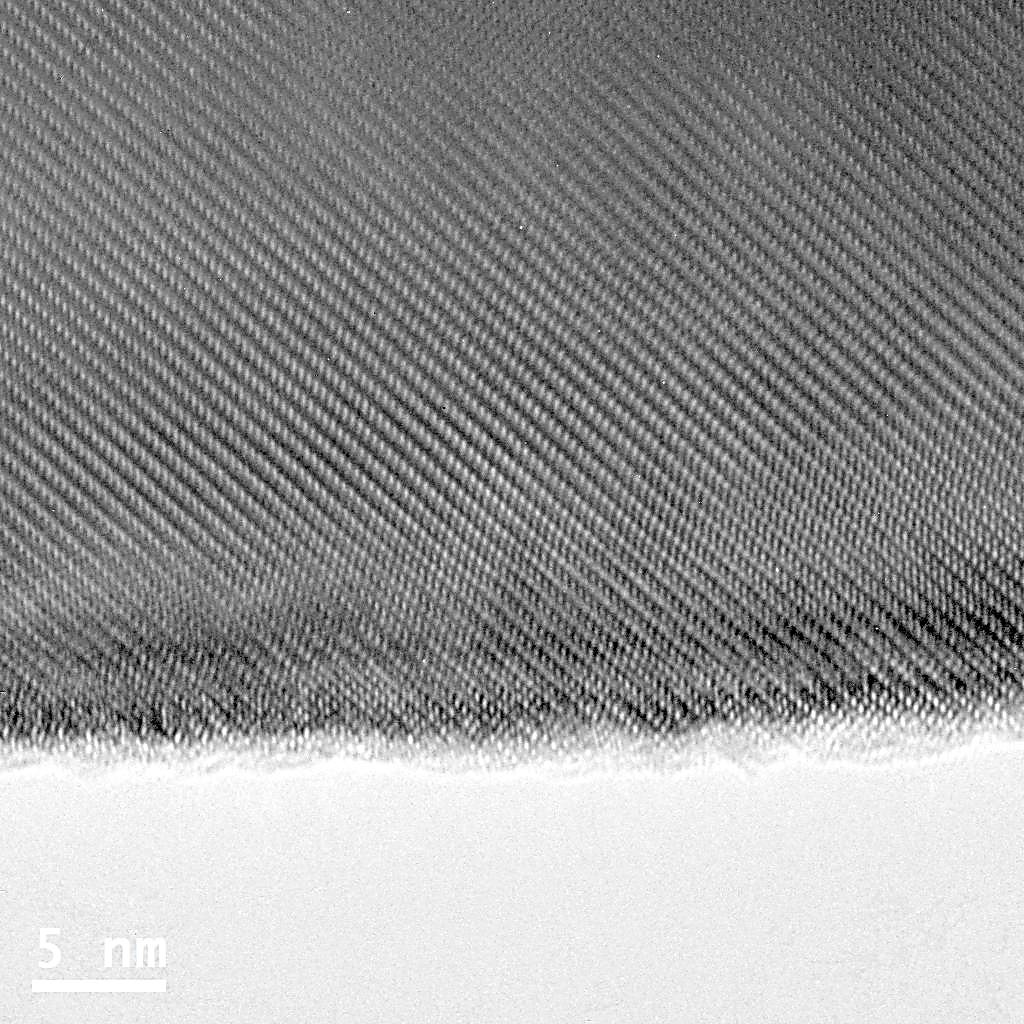


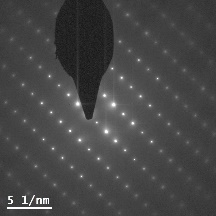

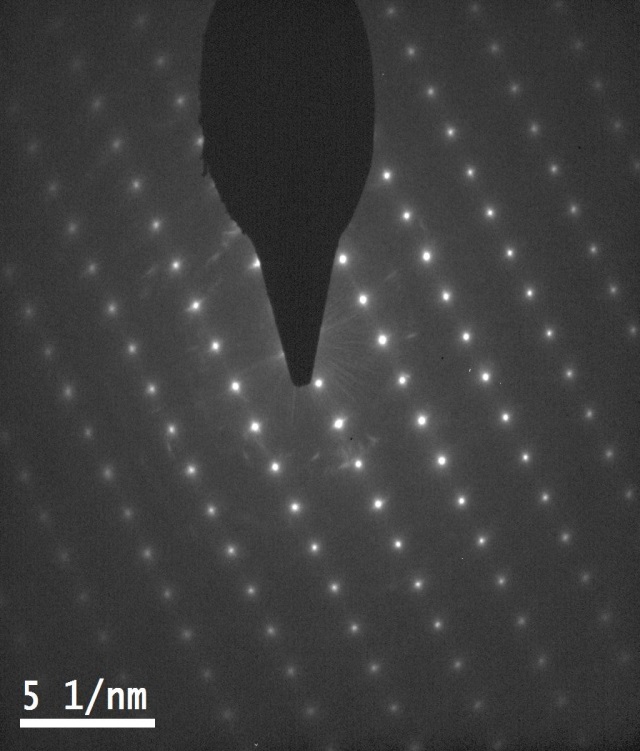


**0001**

**10-01**

**0001**

**10-01**


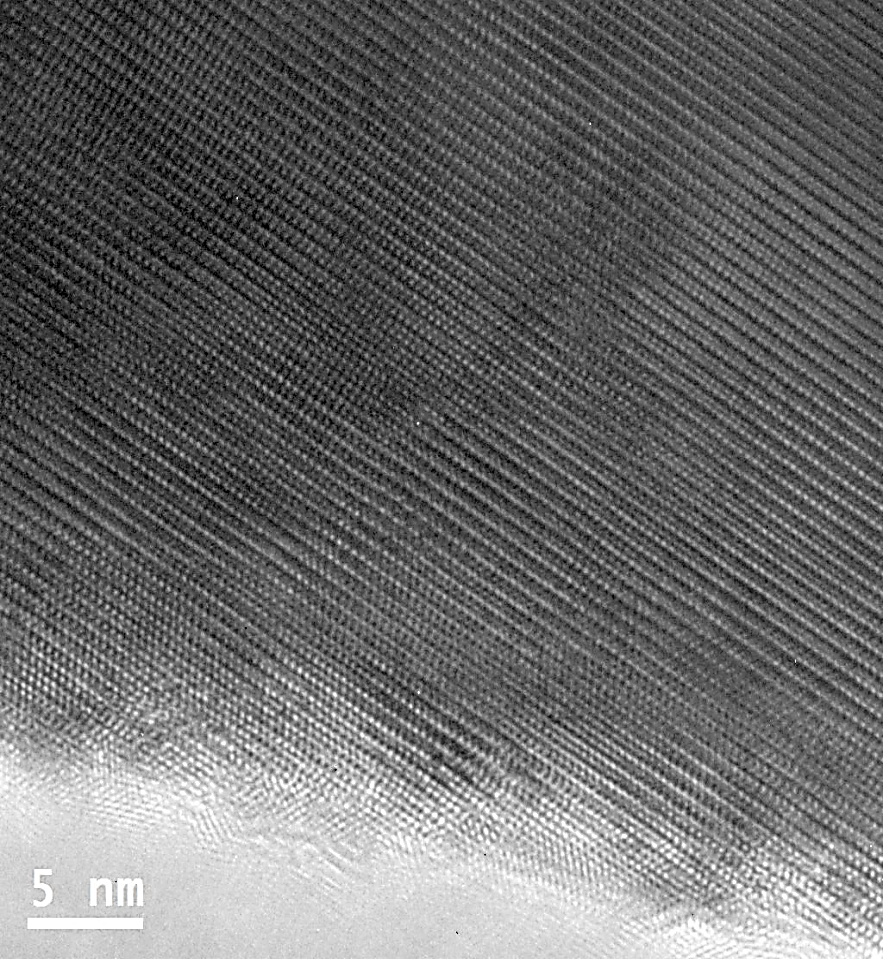

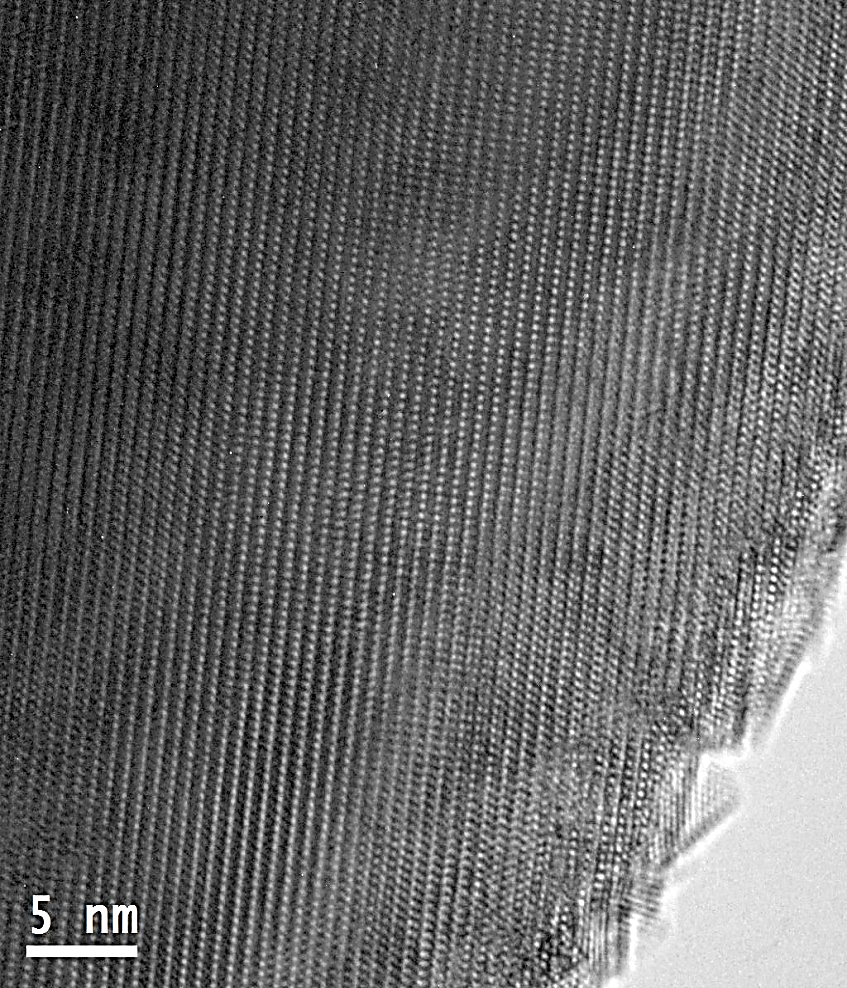


**(d)**

**(c)**


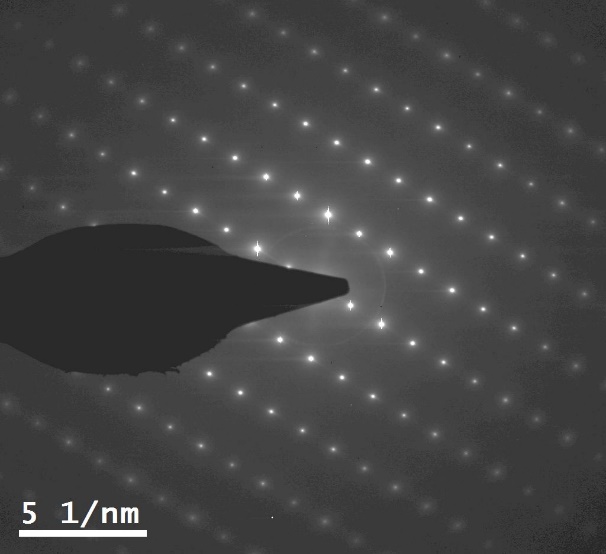

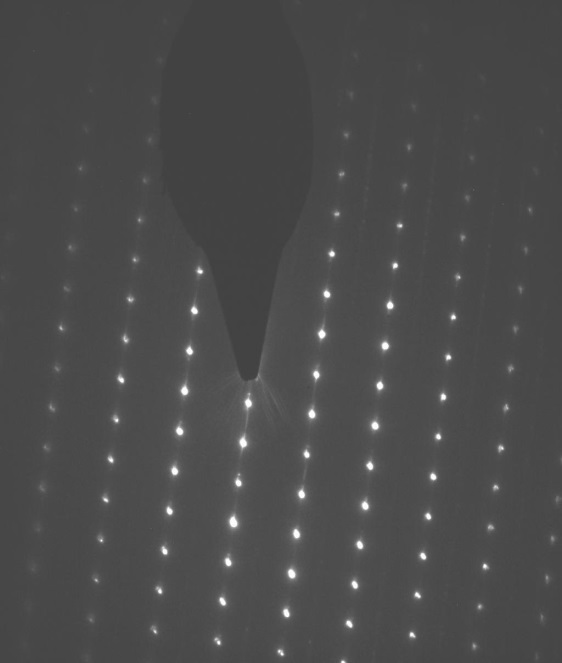


**10-01**

**0001**

**0001**

**10-01**

**FIGURE S2**(a−d)：HRTEM images of the selected NBs and its corresponding SAED pattern depicted as insets (scale bar = 5 nm) for samples A, B, C and D respectively.

**(b)**

**(a)**

**(d)**

**(c)**

**(e)**

**FIGURE S3** (a−e)： XRD patterns of the samples A, B, C, D, and pure CdS nanobelt, respectively. Insets are the zoomed area from 24 to 30^○^ fitted with multi-peak Gaussian function.

**TABLE S1:** Peak shifts and FWHM of major XRD peaks of all the samples (Figure S3).

|  | 2θ^○^ | | |
| --- | --- | --- | --- |
| Pure-CdS | 24.86 | 26.45 | 28.22 |
| CdS:NiF_2_ | 25.02 | 26.70 | 28.38 |
| CdS:NiCl_2_ | 25.10 | 26.76 | 28.44 |
| CdS:NiBr_2_ | 25.32 | 26.98 | 28.66 |
| CdS:NiI_2_ | 25.16 | 26.86 | 28.54 |
|  | **XRD peak FWHM** | | |
| Pure-CdS | 0.113 | 0.116 | 0.137 |
| CdS:NiF_2_ | 0.133 | 0.116 | 0.137 |
| CdS:NiCl_2_ | 0.133 | 0.116 | 0.137 |
| CdS:NiBr_2_ | 0.129 | 0.129 | 0.137 |
| CdS:NiI_2_ | 0.115 | 0.117 | 0.129 |

**(b)**

**(a)**

**(c)**

**(d)**

**FIGURE S4**(a−d)：Raman spectra of samples A, B, C, and D.

**TABLE S2:** List of the Raman frequencies derived from the Raman spectra shown in **Figure S4.**

| Phonon | **1TO** | **E_1_(TO)** | **E_2_(high)** | **1LO** | **2LO** |
| --- | --- | --- | --- | --- | --- |
| Pure-CdS | 215.1 | 239.0 | 255.5 | 303.3 | 604.9 |
| sample-A | 216.2 | 239.7 | 256.6 | 304.2 | 605.4 |
| sample-B | 214.5 | 240.4 | 255.3 | 304.1 | 605.5 |
| sample-C | 213.7 | 237.0 | 255.2 | 304.7 | 605.5 |
| sample-D | 216.3 | 240.7 | 254.0 | 304.6 | 605.5 |

TO, transverse optic; LO, longitudinal optic.

|  |  |  |  |
| --- | --- | --- | --- |

**TABLE S3:** Magnetic parameters (saturation magnetization M_s_, Remanence M_r_, and Coercivity H_c_) of samples A, B, C, and D derived from **Figure 3**.

|  | M_s_ (Am^2^/Kg) | M_r_ (Am^2^/Kg) | H_c_ (Oe) |
| --- | --- | --- | --- |
| CdS:NiF_2_ | 2.42×10^-4^ | 0.42×10^-4^ | 474.9 |
| CdS:NiCl_2_ | 0.69 ×10^-4^ | 0.38×10^-4^ | 1665.1 |
| CdS:NiBr_2_ | 2.38×10^-4^ | 0.82×10^-4^ | 951.3 |
| CdS:NiI_2_ | 2.87×10^-4^ | 1.23×10^-4^ | 1097.2 |

**TABLE S4:** Near bandedge (NBE) emission peak energy derived from the PL spectra shown in **Figure 4**.

| **Samples** | **NBE** |
| --- | --- |
| Pure-CdS | 511.4nm |
| sample-A | 512.9nm |
| sample-B | 517.1nm |
| sample-C | 512.3nm |
| sample-D | 534.4nm |

**FIGURE S5:** The PL spectra of pure CdS NB.

**FIGURE S6**: the absorption spectra of CdS:NiF_2_, CdS:NiCl_2_, CdS:NiBr_2_ and CdS:NiI_2_ NBs by the reflectance mode.




**FIGURE S7:** (a) and (b) are the energy DOS structures of CdS:Ni(left) for Ni-Ni cluster ferromagnetic (FM) and antiferromagnetic coupling(AFM) by Ab initio calculation; (c) contains two energy band structures for FM Ni(II) coupled CdS with spin-up states(left) and spin-down state(right); (d) contains two energy band structures for AFM Ni (II) coupled CdS with spin-up states(left) and spin down state(right); (e) is the DOS structure of CdS+FM Ni2 + I system; (f) shows two energy band structure for FM Ni(II) pair with spin-up (left) and spin-down states. All the above calculations have been carried out based on the procedures proposed by the following references (1. Kresse G and Joubert D, From ultrasoft pseudopotentials to the projector augmented-wave method *Phys. Rev. B* **59** 1758(1999); 2. Blöchl P E, Projector augmented-wave method *Phys. Rev. B* **50** 17953(1994); Perdew J P, Burke K and Ernzerhof M 1996 Generalized gradient approximation made simple *Phys. Rev. Lett.* **77** 3865(1996).

**(a)**

**(b)**

**(d)**

**(c)**

**(f)**

**(e)**

**FIGURE S8:** The lasing profiles of CdS:NiF_2_ (a), CdS:NiCl_2_ (b) and CdS:NiBr_2_ (c) nanobelt under fs pulse excitations at varied fluences.

**TABLE S5:** Pump-fluence dependent lifetimes for λ_em_: 530.9, and λ_em_: 789.3 nm in sample-D.

| **λ_em_: 530.9** | | |
| --- | --- | --- |
| **Pump fluence (kW/cm^2^)** | **τ_1_**  **(ns)** | **τ_2_**  **(ns)** |
| 100 | 0.95 (89%) | 3.72 (11%) |
| 150 | 0.75 (98%) | 3.14 (2%) |
| 180 | 0.62 (93%) | 2.98 (7%) |
| 300 | 0.45 (90%) | 2.91 (10%) |
| 350 | 0.37 (83%) | 1.71 (17%) |
| 410 | 0.28 (72%) | 1.12 (28%) |
| **λ_em_: 789.3** | | |
| **Pump fluence (kW/cm^2^)** | **τ_1_**  **(ns)** | **τ_2_**  **(ns)** |
| 30 | 3.2 (19%) | 142 (81%) |
| 40 | 17.1 (21%) | 131 (79%) |
| 70 | 22.3 (23%) | 103 (77%) |
| 180 | 8.1 (22%) | 23 (78%) |
| 300 | 0.41 (14%) | 10.5 (86%) |
| 350 | 0.35 (11%) | 7.2 (89%) |
| 410 | 0.34 (12%) | 5.3 (92%) |

(a)

(b)

Figure S9(a), Ni and Br dopant concentration dependent PL spectra of CdS:NiBr NBs; (b), Ni dopant concentration dependent PL spectra of CdS:NiI NBs.

**
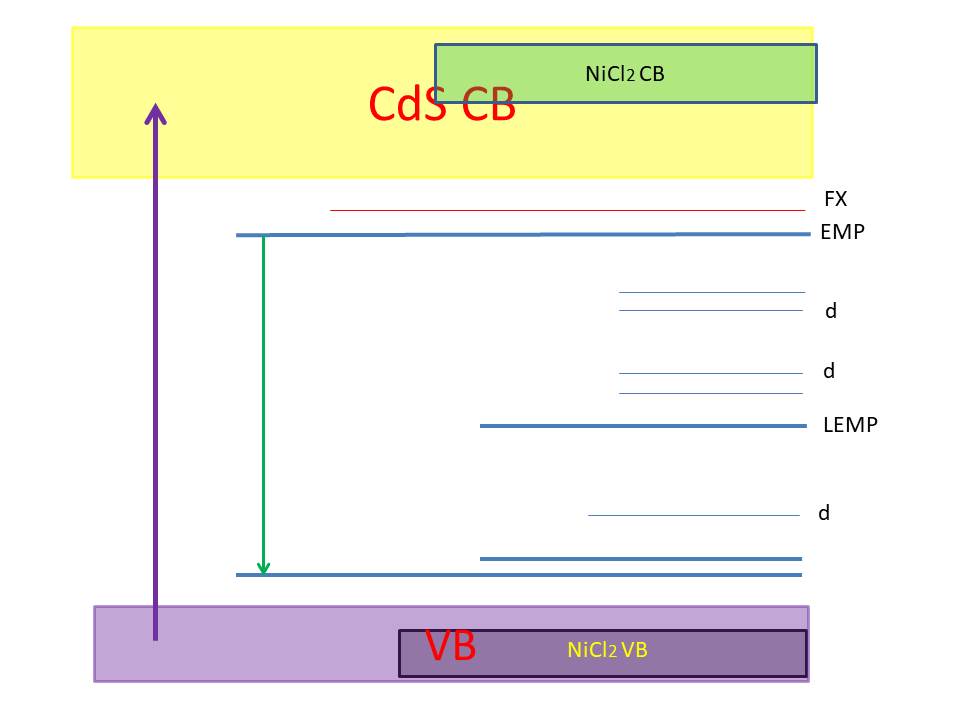

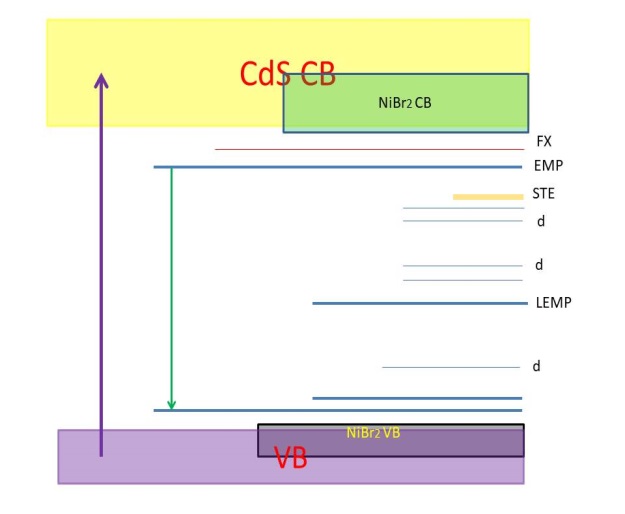
**

1. **(b)**

**FIGURE S10:** The electronic state diagrams of CdS:NiCl_2_(a), and CdS:NiBr_2_ (b) NBs in which the green band represented the doped charge-transfer band Ni-X in CdS NBs.
